# Nanochannels in Fused Silica through NaOH Etching Assisted by Femtosecond Laser Irradiation

**DOI:** 10.3390/ma17194906

**Published:** 2024-10-07

**Authors:** Pasquale Barbato, Roberto Osellame, Rebeca Martínez Vázquez

**Affiliations:** 1 Institute for Photonics and Nanotechnologies, National Research Council (CNR), Piazza Leonardo Vinci 32, 20133 Milan, Italy; roberto.osellame@cnr.it; 2 Physics Department, Politecnico di Milano, Piazza Leonardo da Vinci 32, 20133 Milan, Italy

**Keywords:** NaOH etching, femtosecond laser irradiation, fused silica nanostructuring

## Abstract

Sodium hydroxide (NaOH) is increasingly drawing attention as a highly selective etchant for femtosecond laser-modified fused silica. Unprecedented etching contrasts between the irradiated and pristine areas have enabled the fabrication of hollow, high-aspect-ratio structures in the bulk of the material, overcoming the micrometer threshold as the minimum feature size. In this work, we systematically study the effect of NaOH solutions under different etching conditions (etchant concentration, temperature, and etching time) on the tracks created by tightly focused femtosecond laser pulses to assess the best practices for the fabrication of hollow nanostructures in bulk fused silica.

## 1. Introduction

Fused silica is a glass made from silicon dioxide (SiO_2_) in a non-crystalline (amorphous) form. It is created by melting high-purity silica at extremely high temperatures and then cooling it rapidly to achieve a solid with exceptional properties [1]. It remains transparent across a wide range of wavelengths, from ultraviolet (~200 nm) to infrared (~2500 nm). It can withstand very high temperatures without melting or cracking and exhibits low thermal expansion. Its resistance to most chemicals allows it to endure harsh environments without significant degradation. Despite being brittle, fused silica has good tensile strength and can withstand significant mechanical stress. This unique combination of properties makes fused silica a crucial material for many advanced applications, covering the fields of optics and photonics, the semiconductor industry, telecommunication, and many others [2].

Fused silica consists of silicon atoms bonded to four oxygen atoms, forming SiO_4_ tetrahedra. These tetrahedra are interconnected via shared oxygen atoms called bridging oxygens (BOs), creating a complex, disordered structure with varying Si-O-Si chain angles. Unlike the long-range-ordered crystalline SiO_2_, fused silica can contain non-bridging oxygens (NBOs), resulting from impurities that act as network modifiers [2,3]. Defects such as E’ centers (silicon bonded to three oxygens with an unpaired electron), oxygen deficiency centers (ODCs), and non-bridging hole centers (NBHOCs) can also occur, typically forming during fabrication, gamma irradiation, or femtosecond laser irradiation [4,5,6,7].

When a femtosecond laser beam with wavelengths in the visible or infrared range is focused on fused silica (and other transparent materials), a permanent modification appears [8]. This happens when the local energy is high enough to trigger non-linear absorption processes like multiphoton absorption, tunnel ionization, and avalanche ionization [9], resulting in the formation of an electron plasma, which in a few μs relaxes, leaving the material permanently modified only in the focal volume, while the surrounding material remains unaltered [10]. The characteristics of the final modification depend upon a variety of irradiation parameters, but traditionally, they have been categorized into three groups [11]: a smooth refractive index change (type I); the formation of nanostructures self-organized in planes, called nanogratings (type II); and microexplosions and ablation (type III). Roughly, one can move from one group to another, increasing the deposited energy [11], although, in recent years, this classification has been expanded, e.g., within type II, a new regime has been identified called type X [12,13]. All these regimes have been extensively studied and exploited in applications, the most notable example probably being the inscription of optical single-mode waveguides in various glasses through type I modifications [14].

Additionally, the modified areas may be more easily dissolved than pristine fused silica by a proper chemical agent [15]. In this way, hollow three-dimensional structures can be fabricated by combining femtosecond laser irradiation with subsequent chemical etching. By moving the sample with respect to the laser focus, one can define arbitrary structures ranging from a simple tunnel (obtained by translating the sample in the plane perpendicular to the laser focus) [16] to more complicated structures and 3D shapes [17], with dimensions ranging from a few micrometers to several centimeters. This two-step process, which will be referred to as FLICE (Femtosecond Laser Irradiation followed by Chemical Etching), has made fused silica a precious platform in fields like microfluidics, lab-on-chip, sensoring, and, recently, extreme laser physics [18,19,20,21].

FLICE requires the etching of fused silica, which, as already stated, is a relatively inert material. At room temperature, pristine fused silica can be quickly dissolved only by hydrofluoric (HF) acid-based solutions. HF must react with all four Si-O-Si bonds of the tetrahedra to break them and produce hexafluorosilicic acid [22]. This reaction can be expressed in a simplified way as follows:

SiO_2_ + 6HF → H_2_SiF_6_ + 2H_2_O

In general, the etching rate increases with HF concentration, and various other substances can be added to obtain better control of the etching rate [3]. The efficacy of HF as an etchant for silicon oxides also made it a popular choice for ultrafast laser-processed fused silica. An etching contrast up to 1:100 between modified and unmodified material and etching rates of ∽300 μm/h of the irradiated tracks have been measured [16].

An alternative is represented by an alkaline aqueous solution at hot temperatures. An experimental study on the etching of vitreous silica (97% SiO_2_) by group I hydroxides was conducted by Hooley [23]. He found that sodium hydroxide (NaOH) and potassium hydroxide (KOH) are the most effective in this class of chemical agents, highlighting the strong dependence of the rates on the solution temperature as well as the etchant and water concentration. In FLICE, KOH and, only recently, NaOH are becoming increasingly adopted as etching agents [24,25,26,27]: not only do they show etching rates of the irradiated tracks comparable to HF solutions, but they also provide even higher etching contrasts (~1:300 for KOH and even 1:600 for NaOH) [26]. Moreover, their use represents a safer alternative to HF both for the user and the environment.

In this work, we aim to optimize the use of NaOH as an etchant for submicrometric hollow channels in bulk fused silica, which can be obtained by irradiating under extreme focusing conditions [28]. We studied the evolution of the etching rate and channels’ profiles as a function of NaOH concentration, temperature, and time to provide a clear overview of how these parameters affect the etching procedure.

## 2. Materials and Methods

The substrate chosen for this work was Corning 7980 fused silica, standard grade. The silica was modified with the laser beam generated by an air-cooled femtosecond Yb-doped fiber laser (Satsuma, Amplitude, Pessac, France), emitting 260 fs pulses at a central wavelength of 1030 nm, with a repetition rate fixed at 25 kHz. Before being directed to the inscription stages, the beam’s wavelength was halved using a second harmonic generation in an LBO crystal. (EKSMAOPTICS, Vilnius, Lithuania) A glycerin-immersion 150× objective (Zeiss, Plan-Apochromat, Jena, Germany) with a numerical aperture (NA) of 1.35 focused the beam into the substrate. The objective can move on the *Z*-axis through a pneumatic 1D translational stage (Aerotech, ANT130-035-L-ZS-PLUS, Pittsburgh, PA, USA), while the sample is mounted on a 2D stage (Aerotech, ANT95-50-XY-CMS-ULTRA), guaranteeing movement on the XY plane. The high NA of the objective enables minimum-size features, with the diameter of the focal spot approximately 250 nm, while glycerin immersion was chosen to minimize spherical aberration, as its refractive index (*n* = 1.456 at 23 °C) was very close to that of the substrate (*n* = 1.460). All the inscriptions were made at a depth of 150 μm from the top surface and always in the same direction, perpendicular to the beam polarization. The beam mean power, measured right before the objective lens, was always 0.90 ± 0.05 mW (or ~730 nJ/μm^2^), slightly higher than the non-linear absorption threshold, found at 0.75 ± 0.05 mW (or ~610 nJ/μm^2^). We define such a threshold as the lowest mean power, resulting in an irradiated track successfully etched by the NaOH solution.

Several identical sets of inscriptions were fabricated on two samples, each set consisting of 26 lines spaced 5 μm apart at different scanning velocities (ranging from 2.3 to 6 mm/s). Two lines at the same velocities were inscribed, and the measurements shown are an average over two values. After the irradiation, the lateral facets of the sample were mechanically polished to avoid possible edge effects, and only at that point were the samples cut with a diamond-wire saw to obtain several smaller samples, each one containing only one set of lines to be tested under different etching conditions. With this procedure, it was possible to obtain samples with identical laser processing conditions so that the final result could be attributed only to the etching process.

The chemical etching was performed on an analog magnetic stirrer hot plate, whose functioning was characterized to guarantee temperatures of 35 ± 5 °C, 60 ± 5 °C, and 75 ± 5 °C, directly measured with a thermometer. The samples were immersed in the NaOH aqueous solution only after it had reached the target temperature. NaOH pellets (Sigma-Aldrich, St. Louis, MI, USA) were dissolved in distilled water to obtain three different concentrations: 0.1 M (0.2 g of NaOH in 50 mL of H_2_O), 1 M (2 g of NaOH in 50 mL of H_2_O), and 10 M (20 g of NaOH in 50 mL of H_2_O).

After the etching, the lengths of the tracks were measured using the optical microscope (Nikon Eclipse me600, Tokyo, Japan), while the profiles were imaged using a scanning electron microscope (Thermo Scientific Phenom Pro, Waltham, MA, USA).

## 3. Results

The samples were produced by irradiating fused silica with a femtosecond laser beam at a wavelength of 515 nm and an energy of 36 nJ, close to the non-linear absorption threshold (30 ± 2 nJ), focused by a high numerical aperture (1.35) glycerin-corrected microscope objective (150×). Several identical samples were produced containing lines inscribed with 13 different pulse densities, or Np (number of pulses per μm), scanning from ~4 to ~11 pulse/μm. Polarization was maintained perpendicular to the sample translation direction. As demonstrated in previous work [28], this range corresponds to a situation where tracks are formed by concatenated or slightly overlapping pulses, for which etching rates (in a 1 M NaOH aqueous solution bath, at 65 °C, for 3 h) exceeding 350 μm/h have been found consistently, and submicrometric cross-sections have been demonstrated along the whole channel. For Np < 5, the pulses are delivered spatially separated into the material, while for Np higher than 9, the cumulative absorption of multiple pulses inhibits the etching of the tracks [28]. Here, this inscription regime has been reproduced by fixing the laser repetition rate (25 kHz) and varying inscription velocities (from 6 to 2.3 mm/s). Two lines were inscribed with the same parameters, and all the data presented in the following are averaged. Thereafter, each sample was submerged in a NaOH aqueous solution under different concentration and temperature conditions, and the results are reported in the following.

### 3.1. Etching Behavior with NaOH Concentration

In order to produce nanochannels, we need to maximize the contrast between the etching rates for irradiated and unirradiated fused silica. In fact, the former is responsible for the channel length, while the latter determines the channel width. In reference [23], it is shown that unirradiated fused silica has the highest etching rate at an 8M concentration of NaOH. We thus explored higher and lower concentration values to keep the channel cross-section as small as possible.

We started by measuring the channels’ lengths as a function of NaOH concentration, fixing the temperature at 60 ± 5 °C for 3 h. Four samples were submerged in aqueous solutions containing 0.1 M (0.2 g of NaOH in 50 mL of H_2_O), 1 M (2 g of NaOH in 50 mL of H_2_O), and 10 M (20 g of NaOH in 50 mL of H_2_O). A sample was also submerged in pure water as a reference.

**Figure 1:**
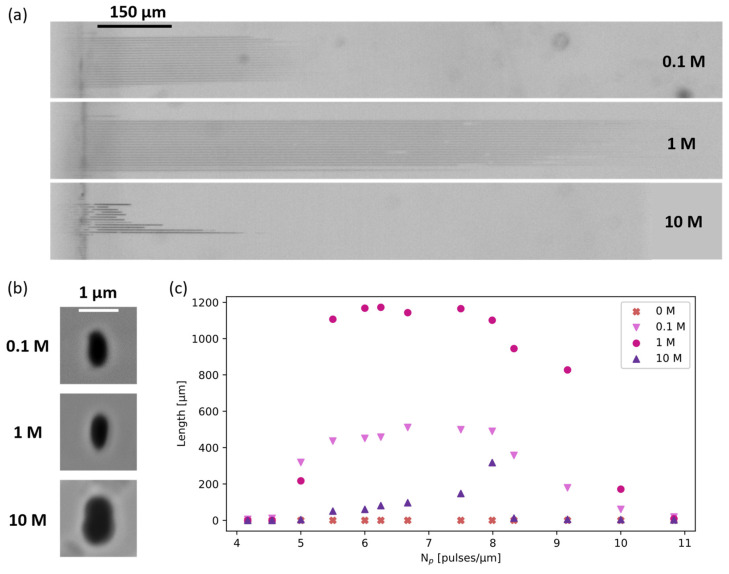
(**a**) Optical microscope top-view of the nanochannels etched at different NaOH concentrations (0.1 M, 1 M, and 10 M). (**b**) SEMs of the N_p_ = 6.7 pulses/μm channels’ cross-sections for the three concentrations and (**c**) length of the etched channels at 0 M, 0.1 M, 1 M, and 10 M NaOH concentrations.

Figure 1 shows both the top view using the optical microscope of all the channels (panel a) and the scanning electron microscope images (SEMs) of a particular, representative channel (panel b) in the middle of the scanned region (Np = 6.7 pulses/μm). Figure 1c reports the measured lengths as a function of Np for the three concentrations. It can be observed that water alone is not able to etch the tracks, while even a small amount of NaOH can effectively dissolve the irradiated material. For the three concentrations explored, we found that longer etching lengths were obtained for the 1 M solution. Looking at the cross-section, there are no relevant differences between the 0.1 M and 1 M tracks, with only the one etched at a 10 M concentration being slightly larger than the others. Thus, 1 M NaOH solution seems to be a good option for obtaining long nanochannels in fused silica. A more complete study on NaOH concentration for irradiated fused silica will be performed in the future.

### 3.2. Etching Dependency on Solution Temperature

Next, we aim to determine how etching behavior changes with solution temperature. Studies on etching of ultrafast laser-processed fused silica using alkaline aqueous solutions typically focus on temperatures ranging from 80 to 90 °C [24,25,26,27]. This is because, at higher temperatures, the reaction kinetics are faster, resulting in higher etching rates. However, more moderate temperatures may increase the selectivity, thus enabling the fabrication of smaller cross-section features. We fixed the NaOH concentration at 1 M and, again, we left the sample submerged for three hours, maintaining the solutions at temperatures ranging from 13 °C to 75 °C.

**Figure 2:**
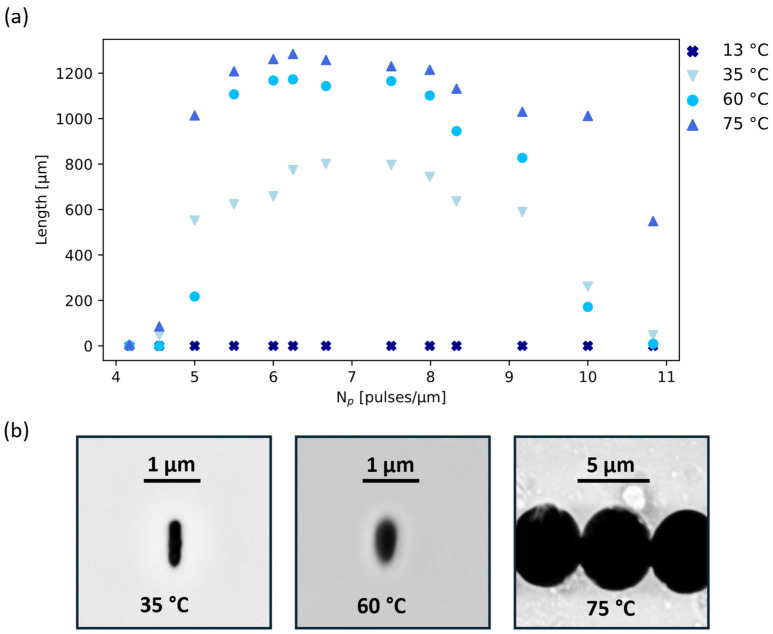
(**a**) Length of the nanochannel after 3 h of etching in a 1 M NaOH aqueous solution as a function of Np for four different solution temperatures. (**b**) SEMs of the cross-section of the channels obtained at 35 °C, 60 °C, and 75 °C solution temperatures.

Figure 2a reports the measured etching lengths for scanned Np at three temperatures, 35 °C, 60 °C, and 75 °C, with temperature fluctuations not exceeding 5 °C. Additionally, a sample was submerged at 13 °C, but no etching was observed. At 35 °C, the channels are etched on average less than at higher temperatures: considering only 6 < Np < 9, the average length of the channel is 751 ± 93 μm at 35 °C, compared to 1116 ± 88 μm at 60 °C and 1230 ± 54 μm at 75 °C. At 75 °C, the slight increase in length is nevertheless accompanied by a significant loss of selectivity, evidenced by the profile shown in Figure 2b. The channel cross-section shows dimensions of a few μm after a 3 h etching due to the increase in the etching rate of the pristine material at this temperature. The channel even connects to the neighbors, irradiated at a distance of 5 μm. On the contrary, at both 35 °C and 60 °C, the channels are submicrometric, even if at 35 °C, the profile is smaller in the horizontal direction (~210 nm) than at 60 °C (~390 nm). The vertical dimensions instead appear comparable for both etching temperatures (~730 nm). We hypothesize that when the channel is small and highly elliptical, the increased etching rate due to a higher temperature may be more effective in the horizontal than in the vertical direction due to a significant difference in the exposed surface. This is visible only for low transversal etching rates, while for higher etching rates (e.g., at 75 °C), this effect rapidly circularizes the cross-section, and then the etching proceeds isotropically, enlarging the channel profile in the same way in both directions.

### 3.3. Evolution in Time of NaOH Etching

**Figure 3:**
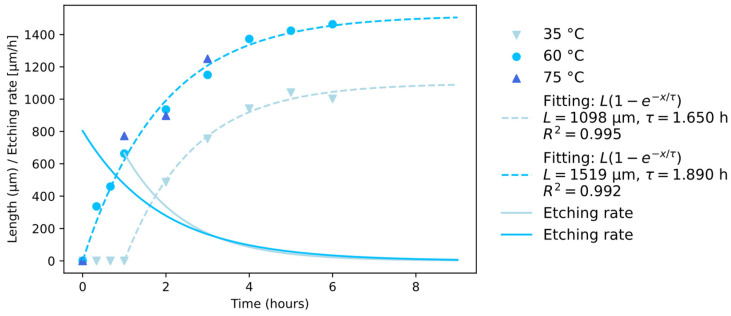
Experimental data, fitting equation, and etching rate describing the time evolution of NaOH etching at different temperatures.

Figure 3 shows the length of the channels as a function of time for the three different temperatures under study. A tendency toward length saturation is evident at both 35 °C and 60 °C. At 75 °C, the evaporation of the solution becomes relevant, causing a change in the etchant concentration, and hence, data acquisition was stopped at 3 h etching time. Additionally, the cross-sections of the channel at this temperature were already a few micrometers large, thus being out of the scope of this work.

At 35 °C and 60 °C, the etching dynamics follow an exponential trend with an equation like L(1 − e^−x/τ^), where L is the saturation length, and τ is the time constant. As expected, at higher temperatures, longer channels can be obtained, but the maximum length will be ~1.5 mm. This saturating behavior implies a non-constant etching rate. The derivative of the fitting function gives a detailed representation of the etching rate, as reported in Figure 3. Initially, the etching rate presents its highest value of L/τ (>800 μm/h at 60 °C), and it then decreases until becoming null after 8 h. Remarkably, at 35 °C, no etching is revealed for the first hour.

**Figure 4:**
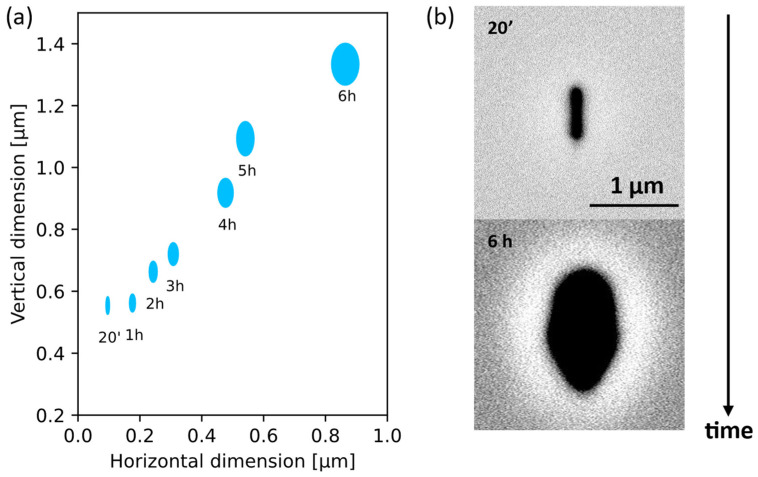
(**a**) Time evolution of the channel dimensions. (**b**) Images of the profile of two tracks etched for 20 min and 6 h, long 317 μm and 1357 μm, respectively.

Alongside the length, we followed the evolution of the channel’s profile, reported in Figure 4. The data shown in panel a) refer to tracks irradiated with Np = 6.7 pulses/μm, and their lengths are the ones reported in Figure 3 for 60 °C. The tracks are elliptical, with starting dimensions of even <100 nm in the horizontal direction (transversal to the laser beam). This ellipticity is probably due to a non-linear effect at the focal spot due to the extreme focusing conditions; circular channels are expected irradiating withlower pulse energies [28]. In the time interval between 20 min and 6 h, there is an enlargement in both directions of ~780 nm (see SEM of the channels’ profiles in Figure 4b). The same tracks present a length of 317 and 1357 μm, respectively.

## 4. Discussion

We presented a systematic study of NaOH etching for the fabrication of nanochannels in fused silica using the FLICE technique. Comparing the maximum etching rates and the track cross-sections after etching, we can conclude that the most efficient condition would be etching at 60 °C as it allows us to obtain longer channels with submicrometer profiles.

Along the tracks, the etching speeds are higher than in the orthogonal direction and rapidly decrease to zero following an exponential trend. In fact, after around three times τ (~5.5 h at 60 °C), the length of the channels completely saturates. On the other hand, we do not observe saturation transversally to the irradiated tracks; rather, we observe continuous enlargement of the section in the horizontal and vertical directions. We can attribute this enlargement to the etching of pristine material, and from the measurement of the cross-section dimensions at different etching times, we can provide an evaluation of the etching rate of the unirradiated material at different temperatures: 0.07 ± 0.01 μm/h at 35 °C, 0.098 ± 0.005 μm/h at 60 °C, and 0.84 ± 0.03 μm/h at 75 °C.

Interestingly, at 60 °C, even a bath of 20 min can result in a few hundred microns of etching length, while the profile does not enlarge too much, making it possible to achieve <100 nm profile channels.

The reasons behind the saturation can be identified in the accumulation of reaction products (sodium silicates) and in the neutralization of the solutions that get into the nanochannel before they reach the non-etched material. Longer etching times and refreshing the solution also proved unsuccessful.

In this work, the irradiation conditions resulted in an elongated voxel, as can be inferred from Figure 4b, and dimensions of <100 nm were measured only in the transversal direction. By optimizing irradiation parameters [28] or writing in the longitudinal direction, more circular profiles can be obtained.

Figure 1 clearly shows that the track’s etching rate does not increase with NaOH concentration. This behavior has already been observed for pristine silica (to a lesser extent and at higher temperatures), where a maximum etching rate was found at 8 M NaOH concentration [23]. This was interpreted by proposing a simplified two-step reaction scheme consisting of water adsorption (1) followed by the reaction of silica with the hydroxyl ion (2):

SiO_2_(s) + 2H_2_O(l) → SiO_2_ · 2H_2_O(s) (1)

SO_2_ · 2H_2_O(s) + 2OH^−^ → [Si(OH)_6_]^2−^ in solution (2)

The role of water is to decrease the screening effect of the highly polarizable oxygen ions towards the silica atoms, which are buried beneath the surface. Thus, a certain optimal water concentration must exist, and if there is too much NaOH in the solution, reaction (1) becomes the bottleneck of the process, explaining the decrease in the etching rate with solution molarity. This two-step reaction also applies to the etching of the irradiated tracks, and therefore, an optimal balance should exist between water and NaOH concentration in the solution. For the values we explored, a 1 M NaOH solution is closest to this optimum. Compared to the striking differences seen in the etched length, the change in the profile dimensions appears almost negligible. In fact, the work cited above reports almost constant etching rates with concentration at temperatures below 70 °C for unmodified fused silica [23].

The dissolution process studied here is heavily dependent on the solution temperature and can also be conducted at very moderate temperatures. Even at 35 °C, channels approaching 1 mm in length have been created. Looking at the profiles of the channels, increasing the temperature sharply increases their size and tends to make them more circular (Figure 2b). An interesting behavior not reported until now for NaOH etching of laser-modified tracks is a delay in the etching start at 35 °C (while the track etching promptly starts at higher temperatures). This period at the beginning of the etching process with no apparent change in the material is known in the literature as the “induction period” [29,30] and is especially relevant at low etchant concentration and temperature.

## 5. Conclusions

The reaction between NaOH aqueous solution and femtosecond laser-processed fused silica differs significantly from that with pristine silica. Structural modifications and the occurrence of defects are the primary causes of the drastic drop in activation energy required for silica dissolution. As a result of this decrease, high etching rates can be achieved even at low temperatures, a condition in which the channel broadening is limited. Additionally, in our regime of inscription parameters, we have observed rapid variability in the etching rate. The etching of the tracks proceeds fast in the first tens of minutes, while the prolonged action of the etchant mainly contributes to channel widening. Thus, variable aspect ratios can be achieved, up to 10^4^ for 1.5 mm long channels at 60 °C. We also highlight the dependence of the etching rates on the etchant concentration, with an optimal value found at 1 M.

These findings shed some light on the behavior of NaOH as an etchant for FLICE and suggest how to optimize the process for creating submicrometer profile-size hollow channels in fused silica. Depending on the desired application and finding the right trade-off between channel length and size, NaOH makes FLICE a technique capable of operating on dimensions smaller than 100 nm, significantly improving the resolution of hollow structures created in bulk fused silica.

## Data Availability

The raw data supporting the conclusions of this article will be made available by the authors on request.

## References

[1] HPFS Product Brochure—Optical Materials Product Information. https://www.corning.com/media/worldwide/csm/documents/HPFS_Product_Brochure_All_Grades_2015_07_21.pdf.

[2] Bourhis E.L. (2008). Glass: Mechanics and Technology.

[3] Zhang X.G. (2001). Electrochemistry of Silicon and Its Oxide.

[4] Munekuni S., Yamanaka T., Shimogaichi Y., Tohmon R., Ohki Y., Nagasawa K., Hama Y. (1990). Various types of nonbridging oxygen hole center in high-purity silica glass. J. Appl. Phys..

[5] Skuja L. (1998). Optically active oxygen-deficiency-related centers in amorphous silicon dioxide. J. Non-Cryst. Solids.

[6] Sun H.-B., Juodkazis S., Watanabe M., Matsuo S., Misawa H., Nishii J. (2000). Generation and Recombination of Defects in Vitreous Silica Induced by Irradiation with a Near-Infrared Femtosecond Laser. J. Phys. Chem. B.

[7] Mishchik K., D’Amico C., Velpula P.K., Mauclair C., Boukenter A., Ouerdane Y., Stoian R. (2013). Ultrafast laser induced electronic and structural modifications in bulk fused silica. J. Appl. Phys..

[8] Davis K.M., Miura K., Sugimoto N., Hirao K. (1996). Writing waveguides in glass with a femtosecond laser. Opt. Lett..

[9] Schaffer C.B., Brodeur A., Mazur E. (2001). Laser-induced breakdown and damage in bulk transparent materials induced by tightly focused femtosecond laser pulses. Meas. Sci. Technol..

[10] Gattass R.R., Mazur E. (2008). Femtosecond laser micromachining in transparent materials. Nat. Photonics.

[11] Poumellec B., Lancry M., Chahid-Erraji A., Kazansky P.G. (2011). Modification thresholds in femtosecond laser processing of pure silica: Review of dependencies on laser parameters [Invited]. Opt. Mater. Express.

[12] Sakakura M., Lei Y., Wang L., Yu Y.-H., Kazansky P.G. (2020). Ultralow-loss geometric phase and polarization shaping by ultrafast laser writing in silica glass. Light Sci. Appl..

[13] Yao H., Xie Q., Cavillon M., Dai Y., Lancry M. (2024). Materials roadmap for inscription of nanogratings inside transparent dielectrics using ultrafast lasers. Prog. Mater. Sci..

[14] Miura K., Qiu J., Inouye H., Mitsuyu T., Hirao K. (1997). Photowritten optical waveguides in various glasses with ultrashort pulse laser. Appl. Phys. Lett..

[15] Marcinkevičius A., Juodkazis S., Watanabe M., Miwa M., Matsuo S., Misawa H., Nishii J. (2001). Femtosecond laser-assisted three-dimensional microfabrication in silica. Opt. Lett..

[16] Bellouard Y., Said A., Dugan M., Bado P. (2004). Fabrication of high-aspect ratio, micro-fluidic channels and tunnels using femtosecond laser pulses and chemical etching. Opt. Express.

[17] Wanie V., Barbato P., Hahne J., Ryabchuk S., Bin Wahid A., Amorim D., Månsson E.P., Trabattoni A., Osellame R., Vázquez R.M. (2024). Ultraviolet supercontinuum generation using a differentially-pumped integrated glass chip. J. Phys. Photonics.

[18] Osellame R., Hoekstra H., Cerullo G., Pollnau M. (2011). Femtosecond laser microstructuring: An enabling tool for optofluidic lab-on-chips. Laser Photonics Rev..

[19] Sugioka K., Cheng Y. (2012). Femtosecond laser processing for optofluidic fabrication. Lab. Chip.

[20] Galli M., Wanie V., Lopes D.P., Månsson E.P., Trabattoni A., Colaizzi L., Saraswathula K., Cartella A., Frassetto F., Poletto L. (2019). Generation of deep ultraviolet sub-2-fs pulses. Opt. Lett..

[21] Ciriolo A.G., Vázquez R.M., Crippa G., Devetta M., Faccialà D., Barbato P., Frassetto F., Negro M., Bariselli F., Poletto L. (2022). Microfluidic devices for quasi-phase-matching in high-order harmonic generation. APL Photonics.

[22] Spierings G.A.C.M. (1993). Wet chemical etching of silicate glasses in hydrofluoric acid based solutions. J. Mater. Sci..

[23] Hooley J.G. (1961). The kinetics of the reaction of silica with group I hydroxides. Can. J. Chem..

[24] Kiyama S., Matsuo S., Hashimoto S., Morihira Y. (2009). Examination of Etching Agent and Etching Mechanism on Femotosecond Laser Microfabrication of Channels Inside Vitreous Silica Substrates. J. Phys. Chem. C.

[25] Ross C.A., MacLachlan D.G., Choudhury D., Thomson R.R. (2018). Optimisation of ultrafast laser assisted etching in fused silica. Opt. Express.

[26] Casamenti E., Pollonghini S., Bellouard Y. (2021). Few pulses femtosecond laser exposure for high efficiency 3D glass micromachining. Opt. Express.

[27] Ochoa M., Roldán-Varona P., Algorri J.F., López-Higuera J.M., Rodríguez-Cobo L. (2023). Polarisation-independent ultrafast laser selective etching processing in fused silica. Lab. Chip.

[28] Barbato P., Osellame R., Vázquez R.M. (2024). Femtosecond Laser Nanomachining of High-Aspect-Ratio Channels in Bulk Fused Silica. Adv. Mater. Technol..

[29] Baklanov M.R., Badmaeva I.A., Donaton R.A., Sveshnikova L.L., Storm W., Maex K. (1996). Kinetics and Mechanism of the Etching of CoSi2 in HF-based Solutions. J. Electrochem. Soc..

[30] Rietig A., Langner T., Acker J. (2023). About determining reliable etching rates and the role of temperature in kinetic experiments on acidic wet chemical etching of silicon. Phys. Chem. Chem. Phys..

